# Attachment, recalled parental rearing, and ADHD symptoms predict emotion processing and alexithymia in adults with ADHD

**DOI:** 10.1186/s12991-015-0082-y

**Published:** 2015-12-02

**Authors:** Marc-Andreas Edel, Susanne Edel, Marie Krüger, Hans-Jörg Assion, Georg Juckel, Martin Brüne

**Affiliations:** Department of Psychiatry, LWL University Hospital, Ruhr University Bochum, Alexandrinenstraße 1-3, 44791 Bochum, Germany; Psychiatry Clinic, LWL Hospital Dortmund, Marsbruchstr. 179, 44287 Dortmund, Germany; Psychiatry Clinic, St. Marien Hospital Eickel, Marienstr. 2, 44651 Herne, Germany

**Keywords:** ADHD, Adults, Emotion processing, Alexithymia, Attachment, Recalled parental rearing behavior

## Abstract

**Background:**

The aim of the aricle is to study the relationship between attachment, parental rearing behavior, and (infant and current) ADHD symptoms with emotion processing and alexithymia in adults with ADHD.

**Methods:**

Attachment, parental behavior, and ADHD variables were tested for predictive value regarding emotion processing and alexithymia in the total sample, the pooled ADHD groups (with inattentive type and combined type, each with *n* = 26) and a control group (*n* = 26). Comparisons were performed between the pooled ADHD groups and the control group, and between the ADHD subtype groups regarding all emotion processing and alexithymia, and attachment-related measures.

**Results:**

Emotion processing/alexithymia parameters were mainly predicted by early or current attachment-related features, and, to a lesser extent, by childhood or current ADHD symptoms, primarily in the ADHD groups.

**Conclusions:**

The findings suggest partly specific and possibly causal relationships between attachment-related features and current emotion processing/alexithymia in adults with ADHD. The results confirm the necessity for further study of the multiple interactions between infant and parental ADHD symptoms, aversive parenting, and attachment with respect to emotional functioning in adult ADHD.

## Background

Attention-deficit/hyperactivity disorder (ADHD) is a common neurodevelopmental disorder in children and adolescents with high pervasiveness into adulthood. The prevalence of ADHD in adulthood is estimated at approximately 4 % [1, 2]. Core domains of ADHD symptomatology are inattention, hyperactivity, and impulsivity [3]. However, Paul H. Wender, a pioneer in diagnosing and treating adult ADHD, introduced three additional symptom categories, which relate to emotion regulation: *affective lability*, *hot temper*, and *stress intolerance* [4]. Emotion regulation can be defined as an individual’s ability to control and modulate emotional responses in order to maintain a motivating level of emotional arousal [5]. Despite emotion dysregulation affects 34–70 % of adults with ADHD and may be a major contributor to impairment, the construct is not conceptualized consensually yet: emotion dysregulation may be, first, a core feature of ADHD, or second, a distinct dimension correlated with ADHD, or third, the combination may be a nosological entity distinct from both conditions [6]. However, the fact that emotion dysregulation is not unique to ADHD, and many individuals with ADHD do not show any emotion dysregulation, suggests that emotion dysregulation and ADHD are rather distinct, albeit associated, categories. Emotion dysregulation in patients with ADHD has shown to be closely related to ADHD core symptom domains, particularly inattention: on the one hand, dysfunctional “top–down” executive control in ADHD may result in impaired allocation of attention to emotionally relevant stimuli. On the other hand, reduced “bottom-up” activation, i.e., orienting toward salient or rewarding emotional stimuli, may also lead to emotion dysregulation (for review of emotion dysregulation in ADHD see Shaw et al. [6]).

However, emotion dysregulation as the central feature of impaired emotion processing in ADHD may have other roots than ADHD-associated dysfunction of attention processes. Attachment, that is, how early mother–child bonding shapes interpersonal behavior in future relationships, has shown to have essential impact on current and future emotion regulation in neurobiological and developmental respect [7]. Also in individuals with ADHD, emotion regulation has presumed to be mediated by poor attachment security [8]. However, empirical research into this association in ADHD is sparse, possibly due to the complexity of multiple interactions of parental as well as infant biological and behavioral factors [9] that may influence both attachment functioning and emotion regulation.

In a previous study with adults with ADHD, we found associations between recalled parental, particularly maternal, ADHD and recalled adverse rearing behaviors on the one hand, and emotion dysregulation and current attachment problems on the other hand [10].

To draw on these findings, the present study focused primarily on possible associations between attachment-relevant experiences in childhood, current attachment features, and infant/current ADHD symptoms on the one side, and current emotion processing facets/alexithymia on the other side.

Emotion processing, comprising emotion identification, regulation, and response [11], has shown to be closely related to the construct of alexithymia [12]. This personality trait that entails problems with identifying, verbalizing, and analyzing feelings, has suggested to be linked to basic emotion processing deficits rather than dysfunction of higher-order emotion regulation dysfunction [13].

In an earlier study on alexithymia, emotion processing, and social anxiety in adults with ADHD, we found close associations between the construct of alexithymia and other emotion processing features. Significant correlations with alexithymia were detected particularly for *acceptance of own emotions* and, to a lesser extent, for *experience of being flooded with emotions* [14]. No such correlations emerged with respect to *experiences of emotion regulation and self*-*control*. This result was in line with the abovementioned finding that alexithymia may be associated with basic emotion processing deficits rather than higher-order emotion regulation dysfunction.

Because of the limited research on emotion processing and alexithymia in adults with ADHD we aimed to target these features in the present study. Possible relationships between attachment and emotion processing were studied in the main subtypes of ADHD, i.e., combined and predominantly inattentive types, vs. control subjects; Finzi-Dottan et al. [15] found that a group of children with combined and predominantly hyperactive-impulsive types of ADHD showed more attachment-related dysfunction than children with the predominantly inattentive type. The children with hyperactive-impulsive ADHD symptoms particularly displayed more parental control, punishing-enmeshed parental behavior (“parental blackmail”), less parental respect for child autonomy, more temperament-emotionality, and more anxious and avoidant attachment styles. Moreover, Maedgen and Carlson [16] reported more emotion regulation problems in children with the combined type than in predominantly inattentive children.

Therefore, assuming different qualities in early child–parent interactions, we expected distinct attachment-relevant experiences and styles and thus different emotion processing features in subtype groups. Moreover, we hypothesized that the ADHD group and the control group would differ significantly in some of the emotion processing, alexithymia, and attachment variables. Above all, we expected that emotion processing and alexithymia were markedly and significantly predicted by attachment measures and (attachment-related) parental rearing behavior. Since we found a significant impact of childhood ADHD symptoms on trauma severity in traumatized adults with ADHD in a recent study [17], we added childhood (besides current) ADHD symptoms as possible predictors of emotion processing features and alexithymia.

## Methods

### Sample

Seventy-eight German adults were included in the study: 26 patients with ADHD-combined type, 26 patients with ADHD-predominantly inattentive type, and 26 healthy controls. Patients with ADHD were randomly recruited from our outpatient unit for adults with ADHD. These patients were distinct from those we had recruited for the abovementioned similar study [9]. The control sample consisted of 22 students of the Ruhr University Bochum and four older siblings of some students. All participants gave full informed consent, and the study has been approved by the Ethics Committee of the Medical Faculty of the Ruhr University Bochum. Gender distribution was balanced between study groups. However, the controls were significantly younger than the patients and had a significantly higher educational level. ADHD subgroups did not differ in basic variables, medication, or comorbidity. For basic data of all groups and comorbidity and current medication data of the subtype groups see Table 1.

**Table 1:** Basic data of all study groups and medication in the ADHD groups

	ADHD-combined type	ADHD-predominantly inattentive type	Healthy controls	Chi-square/*F*/*p*
Gender (m/f)	13/13	14/12	14/12	–/–/0.951^b^
Age (years)	39.1 ± 9.6	38.9 ± 10.6	25.8 ± 3.1	–/21.3/0.000^c^
Education^a^	3.3 ± 1.1	3.1 ± 1.1	4.3 ± 0.5	–/12.4/0.000^c^
Methylphenidate alone	11	6	–	2.185/–/0.139^d^
Antidepressant alone	4	8	–	1.733/–/0.188^d^
Methylphenidate plus antidepressant	4	6	–	0.495/–/0.482^d^
No medication	7	6	–	0.103/–/0.749^d^
Axis-I comorbidity	1.5 ± 1.1	1.6 ± 1.2	–	–/–/0.716^e^
Axis-II comorbidity	1.5 ± 1.2	1.8 ± 1.2	–	–/–/0.350^e^

^a^Education: 2 = secondary school level; 3 = training qualification; 4 = higher education entrance qualification; 5 = university degree

^b^Kruskal–Wallis test

^c^ANOVA

^d^Chi square

^e^ t test

### Assessment of ADHD and axis-I and axis-II comorbidity

To assess childhood ADHD symptoms retrospectively we used the short version of the Wender Utah Rating Scale (German WURS-k) [18]. WURS-k is a self-rating instrument with 25 items, including 4 distractor items (range from 0 to 4 per item), which has a good split-half correlation (*r* = 0.85) and an excellent internal consistency (*α* = 0.91). WURS-k scores at the cutoff of 30 indicate childhood ADHD with a sensitivity of 85 % and a specificity of 76 %. Current ADHD symptoms, i.e., the domains of inattentiveness, hyperactivity, and impulsivity, were examined by means of the ADHD Diagnostic Checklist (ADHD-DC) and the ADHD Self-Rating Scale (ADHD-SR) [19]. These two instruments allow for a combined categorical (ADHD-DC) and dimensional (ADHD-SR) assessment of ADHD according to DSM-IV criteria. The ADHD-SR has excellent retest reliability, good internal consistency, sufficient convergent and divergent validity, and a diagnostic sensitivity of 65–88 % and a specificity of 67–92 % at different cutoff points.

ADHD ratings were performed in the control group as well, but did not reveal any full ADHD diagnoses. Moreover, the SCID I/II [20] for comorbid axis-I and axis-II diagnoses was applied in the ADHD groups. The control group participants were just asked whether they had any lifetime or current mental disorders.

### Assessment of emotion processing and alexithymia

We assessed emotion processing using two self-report instruments. First, we applied the German *Skala zum Erleben von Emotionen*, which can best translated as *Experience of Emotions Scale* (EES) [21]. This instrument comprises a total of 42 items and seven subscales: *acceptance of own emotions*, *experiencing being flooded with emotions*, *experiencing lack of emotions*, *experiencing body*-*related symbolization of emotions*, *experiencing imaginative symbolization of emotions*, *experiencing emotion regulation*, and *experiencing self*-*control*. The EES allows for the assessment of a person’s attitudes toward her or his emotions. It has shown good internal consistency, excellent retest reliability, and sufficient convergent and divergent validity. We excluded the two (body-related and imaginative) symbolization-of-emotions measures and the subscale *experiencing lack of emotions.* The four most suitable EES subscales to serve as dependent variables, covering important facets of emotion processing, were *acceptance of own emotions, experiencing being flooded with emotions, experiencing emotion regulation*, and *experiencing self*-*control.*

Second, we used the 20-item Toronto Alexithymia Scale (TAS-20), which examines difficulties in differentiating and describing emotions [22]. The TAS-20 is a self-report questionnaire with 20 items and a five-point scale per item, which has shown very good internal consistency, good retest reliability, and sufficient convergent and divergent validity. The instrument consists of three dimensions: (1) difficulty identifying feelings, (2) difficulty communicating feelings to others, and (3) externally oriented thinking. We used the TAS-20 total scale, comprising these three dimensions, to assess alexithymia.

### Assessment of attachment features and recalled parenting

First, attachment features were assessed by using the German *Beziehungsspezifische Bindungsskalen für Erwachsene*, which can be translated as *Relationship*-*Specific Attachment Scale for Adults* (RASA), a questionnaire for the assessment of current attachment styles with regard to the mother and the intimate partner [23]. The RASA examines two attachment domains, namely *secure vs. anxious* and *dependent vs. independent*. The instrument has shown to have excellent specificity regarding the attachment partner (mother or intimate partner), good reliability and efficiency, and good convergent and divergent validity.

Second, we applied the German version of the Adult Attachment Scale (AAS), a self-report instrument, which reflects attachment-related attitudes, to measure to what extent a person feels (1) comfortable with closeness and intimacy, (2) comfortable being dependent on others, and (3) anxious about rejection or abandonment [24]. The AAS has shown satisfactory reliability (*α* = 0.72–0.79) and good convergent and divergent validity.

To study recalled parenting, we used the German version (FEE) of the Questionnaire of Recalled Parental Rearing Behavior (QRPRB) [25], originally published as EMBU [26]. The 24-item QRBRB addresses recalled maternal and paternal rearing behavior. It comprises factor-analytically derived dimensions of (1) rejection and punishment, (2) emotional warmth, and (3) control and overprotection by the mother and father, respectively. The QRBRB has shown to have a good reliability (with an internal consistency between 0.72 and 0.89 and Spearman-Brown split-half coefficients between 0.70 and 0.88) and satisfactory validity.

### Statistical analyses

Statistical analyses were performed with SPSS version 22.0 for Mac. We calculated ANOVAs and two-tailed t-tests for independent samples for group comparisons of interval scale data, the Kruskal–Wallis test and Chi-Square tests for group comparisons of nominal scale data, and linear regression models with backward elimination of variables, with emotion processing and alexithymia measures as dependent variables, and scales on attachment, recalled parenting, and (infant and current) ADHD symptoms as independent variables. Moreover, sex, age, education, and group entered the regression analyses as independent co-variables, in order to control for any impact on the dependent variables. Because of multiple comparisons the level of statistical significance was set at *p* < 0.01.

## Results

### Impact of attachment, parenting, and ADHD symptoms on emotion processing and alexithymia

#### Acceptance of own emotions

In the total sample, 22 % of the variance in *acceptance of own emotions* was explained by a regression model, comprising *secure (vs. anxious) attachment in relation to partner*, *education*, and *group* (see Table 2). In contrast, in the pooled ADHD groups, i.e., a combination of the combined type and predominantly inattentive type groups, 65 % of the variance in *acceptance of own emotions* was explained by a model consisting mainly in attachment variables (RASA, AAS) and measures reflecting recalled parental *rejection and punishment* (QRPRB) (see Table 3). No significant regression model was found to explain the variance in *acceptance of own emotions* in the control group.

**Table 2:** Impact of attachment, parenting, and ADHD symptoms on emotion processing and alexithymia in the total sample

Dependent variable (emotion processing)	Independent variables^a^ (attachment, parenting memories, ADHD symptoms, sex, age, education, and group)	*β*/*t*/*p*	ANOVA/*R* ^2^
Acceptance of own emotions (EES)	Secure (vs. anxious) in relation to partner (RASA)	0.25/1.7/0.010	*df* = 3; *F* = 4.8; *p* < 0.01/*R* ^2^ = 0.22
	Education	−0.26/−1.8/0.074	
	Group	0.25/1.7/0.089	
Experiencing being flooded with emotions (EES)	Secure (vs. anxious) in relation to partner (RASA)	−0.20/−2.1/0.045	*df* = 4; *F* = 19.2; *p* < 0.001/*R* ^2^ = 0.61
	Emotional warmth of mother (QRPRB)	0.17/1.7/0.096	
	Childhood ADHD symptoms (WURS-k)	0.60/5.7/0.000	
	Sex	−0.39/−4.3/0.000	
Experiencing emotion regulation (EES)	Secure (vs. anxious) in relation to mother (RASA)	−0.40/−3.1/0.003	*df* = 9; *F* = 5.0; *p* < 0.001/*R* ^2^ = 0.51
	Secure (vs. anxious) in relation to partner (RASA)	0.53/3.8/0.000	
	Depend. (vs. independ.) in relation to partner (RASA)	−0.22/−1.7/0.100	
	Feeling comfortable depending on others (AAS)	−0.33/−2.0/0.057	
	Rejection and punishment by mother (QRPRB)	−0.40/−2.6/0.015	
	Emotional warmth of father (QRPRB)	0.39/2.9/0.006	
	Childhood ADHD symptoms (WURS-k)	0.39/1.9/0.064	
	Current inattentiveness (ADHD-SR)	−0.33/−1.8/0.071	
	Education	−0.26/−1.9/0.063	
Experiencing self-control (EES)	Depend. (vs. independ.) in relation to mother (RASA)	−0.26/−2.2/0.032	*df* = 5; *F* = 7.2; *p* < 0.001/*R* ^2^ = 0.43
	Feeling comfortable with closeness and intimacy (AAS)	0.43/2.8/0.008	
	Current hyperactivity (ADHD-SR)	−0.65/−1.7/0.095	
	Current impulsivity (ADHD-SR)	−0.66/−2.7/0.095	
	Current ADHD symptomatology (total) (ADHD-SR)	1.02/2.3/0.025	
Alexithymia (TAS-20)	Secure (vs. anxious) in relation to partner (RASA)	−0.53/−4.3/0.000	*df* = 3; *F* = 8.1; *p* < 0.001/*R* ^2^ = 0.33
	Anxiety about rejection or abandonment (AAS)	−0.29/−2.1/0.046	
	Emotional warmth of father (QRPRB)	0.31/2.1/0.039	

^a^Collinearity statistics indicated that the independent variables did not explain each other (all variables with a tolerance >0.1 and a variance inflation factor <10)

**Table 3:** Impact of attachment, parenting, and ADHD symptoms on emotion processing and alexithymia in the extended ADHD group

Dependent variable (emotion processing)	Independent variables^a^ (attachment, parenting memories, ADHD symptoms, medication, axis-I/II disorders, sex, age, and education)	*β*/*t*/*p*	ANOVA/*R* ^2^
Acceptance of own emotions (EES)	Secure (vs. anxious) in relation to mother (RASA)	−0.32/−2.4/0.024	*df* = 9; *F* = 5.3; *p* < 0.001/*R* ^2^ = 0.65
	Secure (vs. anxious) in relation to partner (RASA)	0.45/2.9/0.007	
	Depend. (vs. independ.) in relation to partner (RASA)	−0.90/−2.3/0.027	
	Feeling comfortable with closeness and intimacy (AAS)	−0.33/−2.4/0.026	
	Feeling comfortable depending on others (AAS)	−0.27/2.1/0.049	
	Rejection and punishment by mother (QRPRB)	−0.40/−2.6/0.015	
	Rejection and punishment by father (QRPRB)	0.28/2.1/0.046	
	Antidepressant medication	−0.40/−3.0/0.005	
	Education	−0.40/−2.9/0.007	
Experiencing being flooded with emotions (EES)	Secure (vs. anxious) in relation to mother (RASA)	0.29/2.1/0.040	*df* = 5; *F* = 6.4; *p* < 0.001/*R* ^2^ = 0.52
	Depend. (vs. independ.) in relation to partner (RASA)	−0.33/−2.4/0.022	
	Anxiety about rejection or abandonment (AAS)	0.55/3.9/0.001	
	Childhood ADHD symptoms (WURS-k)	0.32/2.4/0.021	
	Current inattentiveness (ADHD-SR)	−0.29/−2.1/0.048	
Experiencing emotion regulation (EES)	Secure (vs. anxious) in relation to partner (RASA)	0.45/3.6/0.001	*df* = 5; *F* = 7.4; *p* < 0.001/*R* ^2^ = 0.55
	Emotional warmth of mother (QRPRB)	−0.27/−2.1/0.045	
	Current hyperactivity (ADHD-SR)	−0.29/−2.1/0.042	
	Antidepressant medication	−0.46/−3.4/0.002	
	Number of axis-II disorders	0.53/3.9/0.000	
Experiencing self-control (EES)	Feeling comfortable with closeness and intimacy (AAS)	0.30/2.0/0.050	*df* = 7; *F* = 3.8; *p* < 0.01/*R* ^2^ = 0.49
	Anxiety about rejection or abandonment (AAS)	−0.32/−1.9/0.068	
	Emotional warmth of mother (QRPRB)	−0.50/−3.5/0.002	
	Childhood ADHD symptoms (WURS-k)	−0.26/1.8/0.078	
	Current hyperactivity (ADHD-SR)	−0.83/−2.1/0.011	
	Current ADHD symptomatology (total) (ADHD-SR)	0.64/2.0/0.055	
	Methylphenidate medication	−0.42/−2.7/0.011	
Alexithymia (TAS-20)	Feeling comfortable with closeness and intimacy (AAS)	0.30/2.0/0.050	*df* = 2; *F* = 9.1; *p* < 0.01/*R* ^2^ = 0.36
	Emotional warmth of mother (QRPRB)	−0.50/−3.5/0.002	

^a^Collinearity statistics indicated that the independent variables did not explain each other (all variables with a tolerance >0.1 and a variance inflation factor <10)

#### Experiencing being flooded with emotions

A model with four variables, one of them concerning ADHD symptoms in childhood (WURS-k), fitted best to *experiencing being flooded with emotions* in the whole sample, and explained 61 % of the variance in the dependent variable (see Table 2). In the pooled ADHD groups, on the other hand, *experiencing being flooded with emotions* was best predicted by several attachment measures, and childhood and current ADHD symptoms had an impact, too (52 % of the variance explained) (see Table 3). Again, no significant regression model was found to explain the variance in *experiencing being flooded with emotions* in the control group.

#### Experiencing emotion regulation

*Experiencing emotion regulation* in the entire sample emerged as predicted best by a set with several attachment parameters, recalled parental behavior variables, ADHD symptoms in childhood and adulthood and educational level (51 % of the variance explained) (see Table 2). With respect to the combined ADHD group, 55 % of the variance of *experiencing emotion regulation* was predicted by five variables (*secure, vs. anxious, in relation to partner*, RASA, *emotional warmth of mother*, QRPRB, *current hyperactivity*, ADHD-SR, *antidepressant medication*, and *number of axis*-*II disorders*) (see Table 3). No significant regression model was found to explain the variance in *experiencing emotion regulation* in the control group.

#### Experiencing self-control

*Experiencing self*-*control* in the total sample was best explained by a model comprising five independent variables in all, which accounted for 43 % of the variance in the dependent variable (see Table 2): *Dependent (vs. independent) in relation to mother* (RASA), *feeling comfortable with closeness and intimacy* (AAS), *current hyperactivity* (ADHD-SR), *current impulsivity* (ADHD-SR), and *current ADHD symptomatology in total* (ADHD-SR).

Regression analysis of the data of the pooled ADHD groups yielded a similar pattern. *Experiencing self*-*control* was strongly predicted by childhood and current ADHD symptoms, and by two attachment-associated variables and *methylphenidate medication*. These seven variables explained 49 % of the variance of the dependent variable (see Table 3). No significant regression model was found to predict *experiencing self*-*control* in the control group.

#### Alexithymia

Finally, *alexithymia*, measured by the TAS-20 total score, was best explained (33 % of the variance) in the whole sample by *secure (vs. anxious) in relation to partner* (RASA), *anxiety about rejection and abandonment* (AAS), and *emotional warmth of father* (QRPRB) (see Table 2). In the pooled ADHD group, 36 % of the variance in alexithymia was explained by *feeling comfortable with closeness and intimacy* (AAS) and *emotional warmth of mother* (QRPRB) (see Table 3). Also in this case, no significant regression model was found to predict *alexithymia* in the control group.

### Comparisons regarding emotion processing, alexithymia, recalled parenting, and attachment between the pooled ADHD group and the control group

Post-hoc Student–Newman–Keuls analyses for multiple comparisons revealed subsets (for *α* = 0.01) with non-significant differences between ADHD subgroups for all comparisons, and significant differences between the ADHD subgroups and the control group for most comparisons. Significant differences (at *p* < 0.01) between ADHD patients and controls were found for *acceptance of own emotions*, *experiencing being flooded with emotions*, but not for *experiencing emotion regulation* and *experiencing self*-*control*. *Acceptance of own emotions* was significantly lower (*df* = 2; *F* = 6.6; *p* < 0.01), and *experiencing being flooded with emotions* was significantly higher (*df* = 2; *F* = 13.6; *p* < 0.001) in the pooled ADHD group. Moreover, ADHD and control groups differed significantly in *secure (vs. anxious)* in relation to the mother or the partner, respectively (RASA), but not in *dependent (vs. independent)* in relation to the mother or the partner, respectively. Furthermore, significant group differences emerged with respect to all three AAS variables (*feeling comfortable depending on others*, *feeling comfortable with closeness and intimacy*, and *anxiety about rejection or abandonment*) and in the TAS-20 total score.

### Comparisons between ADHD subgroups

Apart from the expected differences regarding symptoms of hyperactivity and impulsivity, no significant differences between ADHD subgroups were found with respect to gender, age, education, medication, and comorbidities, as mentioned above. Moreover, no differences emerged for emotion processing (EES subscales), alexithymia (TAS-20), attachment, (RASA, AAS), or recalled parenting (QRPRB), respectively.

## Discussion

The present study sought to investigate the impact of parenting behaviors, attachment features, and (infant and current) ADHD symptoms on emotion processing and alexithymia in the whole study sample, the pooled ADHD group (that is the combination of subtype groups) and the control group. Moreover, the study was concerned with differences in recalled parental rearing behavior, attachment traits, emotion processing, and alexithymia between the pooled ADHD group and a healthy control group, and between the ADHD subtype groups.

As our main finding, emotion processing and alexithymia measures in the whole sample and particularly in the pooled ADHD group, but not in the control group, were predicted, in part, by attachment-related variables, and, to a lesser extent, by childhood and current ADHD symptoms and other variables.

On closer examination, the acceptance of own emotions was predicted primarily by features of anxious and dependent attachment and recalled parental rejection and punishment in the pooled ADHD group, to a lesser extent in the total group, but not in the control group. This suggests an ADHD-specific relationship between attachment-related facets including early aversive parental behavior on the one hand and *acceptance of own emotions* on the other. Furthermore, the impact of the covariate *education* on the acceptance of own emotions was more pronounced with respect to the pooled ADHD group than regarding the total sample—possibly because of a weak self-esteem of the ADHD patients related to lower graduation. Notably, ADHD symptoms did not have any impact on the acceptance of own emotions.

*Experiencing being flooded with emotions* was associated with several attachment-related measures, both in the pooled ADHD group and the total group. However, also ADHD symptoms in childhood and, with respect to the pooled ADHD group, current symptoms of inattention, had an impact on the feeling to be flooded with emotions. Again, the relationship between attachment features and the emotion processing measure appeared to be closer in the ADHD group than in the whole sample, and no such relation was found in the control group, which also might indicate an ADHD-specific association.

*Experiencing emotion regulation* was also predicted by both attachment and ADHD parameters. However, the impact of attachment on emotion regulation did not stand out as ADHD-specific, which is in accordance with findings that suggest a general association between the two domains. Empirical research has demonstrated overall that children who have secure attachment histories with their mothers regulate their emotions more effectively than do children with insecure histories [27].

The prediction of *experiencing self*-*control* emerged to be predominantly explained by current ADHD symptomatology, which was the case in the pooled ADHD group as well as in the whole sample, but not in the control group. In the pooled ADHD group, furthermore, childhood ADHD symptoms and methylphenidate medication had an impact on *experiencing self*-*control*.

Alexithymia as a dependent variable was exclusively explained by attachment parameters and attachment-related measures (parental emotional warmth), but not by ADHD symptoms, in the total sample as well as in the ADHD group, but not in the control group. This might, once more, indicate a specific relationship between attachment and alexithymia in adults with ADHD. However, despite missing associations between ADHD symptoms and alexithymia in this study, impaired cognitive and particularly verbal abilities generally may contribute to alexithymia in ADHD.

Per group comparison, we expectedly found significant differences between the pooled ADHD group and the control group with respect to the most of the emotion processing/alexithymia and attachment-related measures. In terms of emotion processing, particularly *acceptance of own emotions* was significantly lower, and *experiencing being flooded with emotions* was significantly higher in the pooled ADHD group than in the control group. Increased experiencing being flooded with emotions in the ADHD group corresponds to Wender’s abovementioned additional symptom domains of ADHD (affective lability, hot temper, and stress intolerance) [4]. Empirical research in adults with ADHD has shown that emotion regulation via acceptance of sadness, contrary to suppression, appeared to allow faster, and prolonged recovery from the experience being overwhelmed with emotion [28]. This finding supports clinical relevance of emotional acceptance in adults with ADHD, suggesting an inverse relation of acceptance of emotions and the experience being flooded with emotions. In fact, “mindful” acceptance of negative experiences is trained as a basic coping strategy in “third wave” behavior therapies for adults with ADHD [29, 30].

Since most patients with ADHD report an everyday struggle for control in various situations and efforts to regulate their emotions, it was counterintuitive for us that we did not find any significant group differences with regard to *experiencing emotion regulation* and *experiencing self*-*control*. This finding might be owed to a leveling between groups through lower introspection or awareness of deficits and thus exaggeration of these rather meta-cognitive/mentalizing-dependent aspects and skills in the ADHD patients. On the other hand, the unexpected effect may be explained by overstatement of own emotion regulation and self-control capabilities as a defense strategy to protect self-esteem against critical or challenging phrasing in the questionnaire concerning profound deficits in ADHD.

Alexithymia, measured by the TAS-20 total score, was significantly higher in the ADHD group. This finding contrasts with our results from an earlier study, which yielded TAS-20 scores in adults with ADHD that were similar to values in general population samples [14].

With respect to attachment-related features, the pooled ADHD group and the control group differed significantly in all variables concerning recalled parental rearing behavior and in the actual attachment measures (including secure/anxious attachment in relation to the mother and the partner)—except dependent/independent attachment style in relation to the mother and the partner, respectively. The finding of significantly less attachment security/more attachment anxiety in the ADHD subtype groups is supported, in part, by the results of Koemans et al. [31]. The authors found a rate of only 18 % in a sample of 84 adults with ADHD who were securely attached (compared with prevalence rates of about 60 % in epidemiological samples with a secure base of attachment). Moreover, they reported a *fearful* attachment style as the main domain (44.4 %), followed by *preoccupied* (27.2 %) and *dismissive* (9.9 %) styles. However, the preoccupied attachment style, which is also denoted as an *enmeshed* or *ambivalent* style, typically involves dependent behavior in relationships [32–34], a trait for which no group difference was found in our study.

However, we did not find any differences between the two ADHD subtype groups regarding emotion processing, alexithymia, and attachment-related measures. This was an unexpected result because, following the findings of Finzi-Dottan et al. [15] and Maedgen et al. [16] in children with ADHD, we assumed that the combined-type patients (with pronounced hyperactivity and impulsivity) would recall more adverse parental behavior like controlling or punishing, and display more attachment dysfunction and emotion regulation problems. However, these results pertain to children, and the situation in adults with ADHD might be more balanced between subtypes.

The present study has several limitations. For one thing, the control group was not well matched. Second, the study exclusively relied on subjective report and recall in adults with ADHD, so it cannot be ruled out that the lack of objective information biased the evaluation to considerable degree. Third, the WURS-k, which was used to assess overall ADHD symptoms in childhood, cannot distinguish between the main ADHD symptom domains. Therefore, information whether infant and current ADHD symptoms have comparable impact on EP was not accessible.

## Conclusions

However, the results do suggest that (adverse) parental rearing behavior and attachment features and, to a lesser extent, childhood and current ADHD symptomatology might, in part, specifically underlie emotion processing and alexithymia in adults with ADHD. Counterintuitively, the pooled ADHD group and the control group did not show any difference concerning experiencing emotion regulation and self-control, and regarding a dependent attachment style. Moreover, the two ADHD subtype groups did not differ in any of the emotion processing/alexithymia and attachment-related features. The results corroborate the necessity for further study of the multiple interactions between infant and parental ADHD symptoms, aversive parenting, and attachment with respect to emotional functioning in adult ADHD.
